# Establishment of a prediction model and immune infiltration characteristics of atherosclerosis progression based on neutrophil extracellular traps-related genes

**DOI:** 10.1590/1414-431X2024e13639

**Published:** 2025-03-03

**Authors:** Yuan Gao, Lele Hui, Gang Dou, Xiaoying Chang, Yue Tang, Hao Liu, Zebiao Xu, Bing Xu

**Affiliations:** 1 Xi'an International Medical Center Hospital, Xi'an, China; 2 No. 215 Hospital of Shaanxi Nuclear Industry, Xianyang, China; 3 Heilongjiang University of Chinese Medicine, Harbin, China; 4 Affiliated Hospital of Shaanxi University of Chinese Medicine, Xianyang, China; 5 Chenggu County Hospital of Traditional Chinese Medicine, Hanzhong, China

**Keywords:** Atherosclerosis progression, Machine learning, Prediction model, Immune infiltration, Real-time quantitative PCR

## Abstract

Neutrophil extracellular traps (NETs) are a novel regulatory mechanism of neutrophils, which can promote endothelial cell inflammation through direct or indirect pathways and play a crucial role in the occurrence and development of atherosclerosis (AS). This study aimed to explore the mechanism of NETs in AS progression using bioinformatics methods. We acquired datasets from Gene Expression Omnibus (GEO) and Kyoto Encyclopedia of Genes and Genomes (KEGG) and used Weighted Gene Co-expression Network Analysis (WGCNA) to identify communal genes shared by NET-related genes. Gene Ontology (GO) and KEGG enrichment analyses were conducted. Machine learning algorithms were used to identify hub genes, then protein-protein interaction (PPI), CO-expression network construction, nomogram model building and validation, and immune infiltration analysis were performed. Data were verified by qPCR. Four datasets related to AS progression were included. Module genes shared 27 genes with NRGs. Pathways related to immune regulation, leukocyte migration, and others were identified. Machine learning revealed SLC25A4 and C5AR1 as hub genes. SLC25A4 and C5AR1 were confirmed to have predictive value for intraplaque hemorrhage (IPH), advanced AS plaques, ruptured plaques, and unstable plaques. These pathologic changes are closely related to AS progression and are the main contents of AS progression. Immune infiltration analysis revealed 4 immune cells associated with IPH, among them resting dendritic cells, which were closely related to SLC25A4. In qPCR validation, SLC25A4 and C5AR1 were shown to be consistent with the bioinformatic analysis results. These findings provided novel insights into the molecular characteristics of NRGs and potential therapies for AS progression.

## Introduction

Atherosclerosis (AS) is a chronic inflammatory disease of the arterial wall, presenting as a progressive disease process. It is characterized by initial dysfunction of endothelial cells and vascular smooth muscle cells, followed by lipid accumulation in the arterial wall, persistent inflammation (1), and ultimately leading to intraplaque hemorrhage (IPH), plaque rupture, and thrombosis, also known as formation of unstable plaques. The disease encompasses a range of cardiovascular and cerebrovascular disorders, such as stroke, ischemic heart disease, and peripheral vascular disease (2), contributing to high mortality rates globally. Most acute coronary events are caused by plaque rupture, which is secondary to plaque progression (3). Studies have indicated that IPH and total plaque volume independently increase the risk of recurrent ipsilateral ischemic stroke or transient ischemic attack in patients with mild-to-moderate carotid stenosis (4). However, the mechanism underlying the progression of AS is intricate and tightly linked to immune regulation. Autoantigen-specific adaptive immune responses have been observed in both patients and animal models of AS, playing a significant role in disease progression and atheroprotection (5).

Plaque progression is modifiable. Some studies suggest that by intensifying lipid-lowering therapy, the progression of plaques can be halted and reversed (3). For instance, it can slow down the progression of the overall volume of early plaques, thereby preventing the development of late plaques. For high-risk plaques (i.e. late plaques/unstable plaques), the probability of plaque rupture can be reduced by modifying the plaque morphology, such as reducing the necrotic core volume and increasing the thickness of the fibrous cap (3). But currently, despite the clinical success of statins and PCSK9 antibodies in treatment, diseases stemming from AS remain the leading cause of mortality worldwide (5). Therefore, it is crucial to explore the molecular mechanisms that drive the progression of AS to discover novel clinical treatment strategies.

The formation process of neutrophil extracellular traps (NETs) is known as NETosis, representing a novel cell death program distinct from apoptosis and necrosis (6). This process involves the activation of neutrophils by an inducer through receptors. Following cell flattening and adhesion to the matrix, intracellular particles are activated, resulting in decondensation of nuclear chromatin, disappearance of lobulation, fusion of the granule membrane with the nuclear membrane, and release of chromatin fibers and granule proteins into the extracellular space (6). Multiple studies have indicated that NETs play a critical role in the progression of AS. For instance, NETs activate plasmacytoid dendritic cells (pDCs) in AS lesions, leading to significant synthesis and release of IFN-I, which further aggravates the inflammatory response, increases plaque burden, decreases collagen deposition, deteriorates the internal environment of plaques, and promotes the progression of AS (7,8). NETs activate macrophages and promote the oxidation of low-density lipoprotein (LDL) into ox-LDL, leading to the development of foam cells and sustaining the progression of atherosclerosis. PAD4-dependent NETs produced by macrophages (METs) and the release of extracellular traps are closely related to the evolution of atherosclerotic plaques. In addition, NETs-related proteins can damage smooth muscle cells and accelerate plaque rupture (8,9). Franck et al. (10) also reported that inhibiting PAD4, a key enzyme for NETs generation, can reduce the plaque area in ApoE/AS mice and improve carotid artery thrombosis.

Overall, the mechanisms underlying the harmful effects of NETs on AS progression have been identified and mainly include the induction of endothelial cell dysfunction and apoptosis, the activation of dendritic cells and macrophages, the recruitment of immune cells, the promotion of autoantibody production, and the acceleration of thrombosis. Among them, the direct effects of NETs on endothelial dysfunction mainly involve DNA, histones, myeloperoxidase (MPO), matrix metalloproteinases (MMPs), serine proteases, and antibacterial peptide LL37 in NETs. Indirect effects mainly involve NLRP3-mediated inflammatory pathways, interleukin-8 (IL-8) pathways, antineutrophil cytoplasmic antibody (ANCA) pathways, and antibacterial peptide (LL37)-pDCs pathways (11). In summary, NETs are regarded as a crucial factor in the progression of AS, and targeting NETs may offer novel strategies for the treatment of AS and prevention of AS-related diseases.

With significant advancements in gene microarray technology, researchers can now rapidly assess the expression levels of thousands of genes. This advancement aids in enhancing our understanding of the genetic basis of disease etiology. Consequently, this study utilized bioinformatics tools to investigate the molecular characteristics of NETs-related genes (NRGs) in the progression of AS, and the results were verified by experiments. The objective was to identify potential biomarkers (including the genome and immune cells) that could improve the prediction and management of AS progression.

## Material and Methods

### Data source and processing

Gene Expression Omnibus (GEO) was produced by the NCBI microarray and high-throughput sequencing database (http://www.ncbi.nlm.nih.gov/geo) (12). Gene datasets were screened using “atherosclerosis” as the keyword according to strict inclusion and exclusion criteria. Inclusion criteria were: 1) must be relevant to the progression of atherosclerosis; 2) being a test specimen of human tissue; 3) must contain test specimens from both the case and control groups. Exclusion criterion was: cannot provide processed or raw data that can be used for reanalysis. Finally, four gene datasets related to the progression of AS were obtained from the GEO after screening and comparison: GSE163154, GSE28829, GSE41571, and GSE120521 (13). The GSE163154 dataset consisted of 27 samples with IPH and 16 samples without IPH. The GSE28829 dataset comprised 16 advanced AS plaques and 13 early AS plaques. The GSE41571 dataset comprised 5 ruptured plaques and 6 stable plaques. The GSE120521 dataset comprised 4 unstable plaques and 4 stable plaques. The GSE163154 dataset served as the training set, while the merged set of the four datasets served as the validation set.

The above datasets were normalized using the “Z-Scores” method. NRGs were obtained from the Kyoto Encyclopedia of Genes and Genomes (KEGG) repository (14).

### Weighted gene correlation network analysis (WGCNA)

This study utilized the “WGCNA” package in R software to investigate the association between genes and phenotypes through the construction of a gene co-expression network (15). Based on GSE163154, WGCNA was employed to identify significant modules associated with IPH and construct a visually represented gene network. Initially, the same gene names were averaged by column using the R “aggregate” function, and we excluded 50% of the genes with the lowest median absolute deviation (MAD). Then, we computed Pearson correlation matrices for all gene pairs and generated weighted adjacency matrices using the average linkage method and weighted correlation coefficients. Subsequently, we calculated adjacency using the “soft” threshold power (b) and transformed it into a topological overlap matrix (TOM). To cluster genes with comparable expression profiles into modules, we conducted average linkage hierarchical clustering using TOM dissimilarity measures and a minimum module size of 100. Lastly, we calculated the dissimilarity among characteristic genes within each module, identified the module dendrogram's cut lines, and merged multiple modules when necessary.

### Functional annotation and pathway enrichment of communal genes

Subsequently, we analyzed the expression patterns of the communal genes. Based on the results of WGCNA, we identified significant modules related to IPH. Then, we intersected these module genes with NRGs to identify communal genes, and communal genes were visualized through Venn diagrams. Furthermore, using the R package “clusterProfiler” (16), we conducted Gene Ontology (GO) and KEGG enrichment analyses on these communal genes (17,18).

### Identification of NETs-related hub genes

Three machine learning algorithms, namely least absolute shrinkage and selection operator (LASSO), support vector machine (SVM), and random forest (RF) were employed. The R packages “glmnet” (19), “e1071” (20), “care” (21), and “random forest” (22) were utilized to identify hub genes among the communal genes. LASSO logistic regression selects variables with the lowest probability of classification error (23). The SVM technique establishes a hyperplane in the feature space that maximizes the margin between negative and positive instances (24). RF represents a collection of independent decision trees that integrate machine learning techniques for regression or clustering predictions (25). The optimal lambda value for LASSO regression was determined through 10-fold cross-validation with 10 resampling iterations. Furthermore, the performance of SVM and RF was assessed using 10-fold cross-validation. Hub genes associated with AS progression were selected by identifying the overlapping genes among the three algorithms.

### PPI and co-expression network construction

Based on the above machine learning algorithm, we have ultimately confirmed the hub genes. Protein-protein interaction (PPI) networks possess the capability to identify central protein genes and uncover protein interactions. The PPI network of the common genes and hub genes (http://string-db.org) was constructed using the STRING database and visualized with Cytoscape when the composite score of interaction exceeded 0.4 (26,27). Furthermore, the online software GeneMANIA (https://genemania.org/) was employed to construct the co-expression network of hub genes (28).

### Construction and validation of a prediction model

Based on the R package “rms”, hub genes were utilized to conduct multivariate logistic regression analysis to develop nomogram models for IPH (GSE163154) and “merged set of the four datasets” (29). When building the model of “merged set of the four datasets”, we adopted the random sampling method, using random representative samples for simulation analysis. Using these pathological changes as evidence and reference for predicting the progression of AS is indeed valuable, given their close association with the disease and their status as the main components of AS progression. The “total number of points” represents the cumulative score assigned to the aforementioned predictors, with each predictor having an associated point value. The receiver operating characteristic (ROC) curve and the corresponding area under the curve (AUC) were calculated using the training (GSE163154) and validation (merged set of the four datasets) sets to assess the diagnostic performance of the nomogram. The ROC curve was evaluated using the “ROC” package in R (30). In addition, decision curve analysis (DCA) and clinical impact curve (CIC) were used to evaluate the accuracy and utility of the diagnostic model.

### Analysis of immune infiltration

Using the CIBERSORT algorithm, we analyzed the proportions of 22 immune cell subsets in both IPH and control samples obtained from GSE163154 (31). Furthermore, violin plots were created using the ggplot2 package to effectively visualize and compare the variations in immune cell populations between IPH samples and control samples. We conducted Pearson correlation analysis between the hub genes and infiltrating immune cells using the ggstatsplot package (32), and the results were then visually represented using the ggplot2 package (33).

### ApoE mouse models

Three 16-week-old ApoE male mice were used as the early AS plaques group and three 19-week-old male ApoE mice were used as the advanced AS plaques group. The aortic arch is one of the common sites of AS progression, and its structural characteristics and hemodynamic specificities make it vulnerable to atherosclerotic impacts. By studying the aortic arch, we can directly observe and understand the pathological process of AS. Secondly, the spleen plays a crucial role in the immune system, participating in the regulation and coordination of immune responses, and also influencing the formation and release of NETs. By studying the spleen, we can gain a deeper understanding of the role of NETs in the progression of AS. Therefore, we have chosen the aortic arch and spleen of mice as the experimental materials.

After these mice inhaled carbon dioxide, the following procedures were performed on each mouse: incision of the skin along the midline of the lower abdomen, thoracotomy to open the chest cavity, resection of the sternum, and dissection and preparation of the thoracic aorta and spleen. Subsequently, the thoracic aorta and spleen were gently removed, rapidly frozen in liquid nitrogen, and then stored in a -80°C freezer for preservation. This study was approved by the Ethics Committee of SPF(Beijing) Biotechnology Co., Ltd. (AWE202404010).

### Real-time quantitative polymerase chain reaction

Total RNA was extracted from the frozen aortic tissue and spleen tissue of each mouse using the TRIzol reagent (Sevier Biotechnology Co., LTD., China). Nanodrop 2000 was used to detect the concentration and purity of RNA. After the instrument blank was set to zero, 2.5 μL of the RNA solution to be tested was placed on the detection base, the sample arm was lowered, and the absorbance value was detected using the computer software. Excessive concentrations of RNA were diluted in appropriate proportions to achieve a final concentration of 200 ng/μL. Subsequently, RNA was reverse transcribed into cDNA using a reverse transcription kit and used as a template for subsequent qPCR experiments. The primer sequences are listed in Supplementary Table S1, and the primers were created by Sangon Biotech (China). Real-time fluorescence quantitative PCR was performed by qPCR instrument, and GAPDH served as the internal reference gene for the PCR data.

## Results

### Weighted gene co-expression network analysis

**Figure 1:**
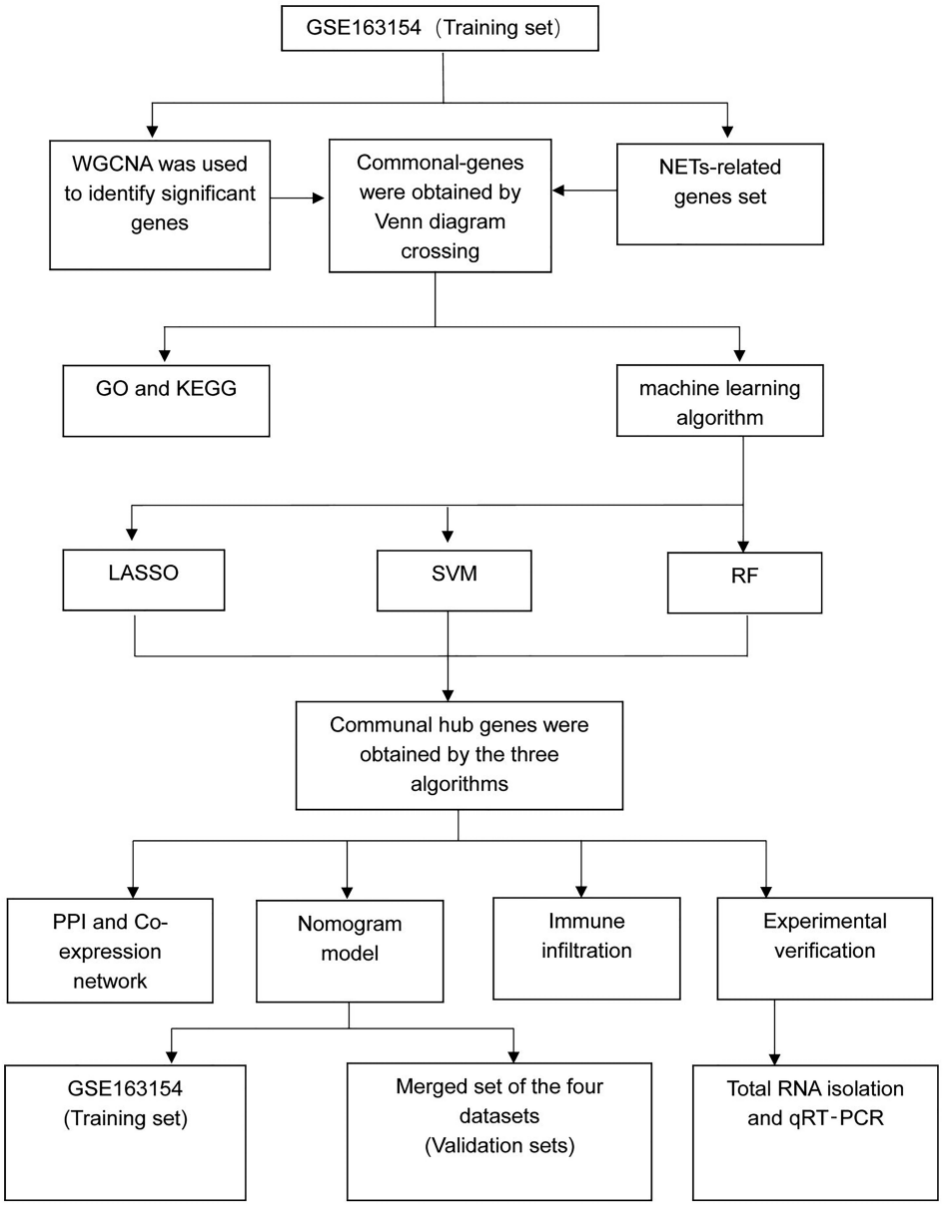
Study flow chart. WGCNA: Weighted gene co-expression network analysis; NETs: neutrophil extracellular traps; GO: Gene oncology; KEGG: Kyoto Encyclopedia of Genes and Genomes; LASSO: least absolute shrinkage and selection operator; SVM: support vector machine; RF: random forest; qPCR: real-time quantitative PCR.

**Figure 2:**
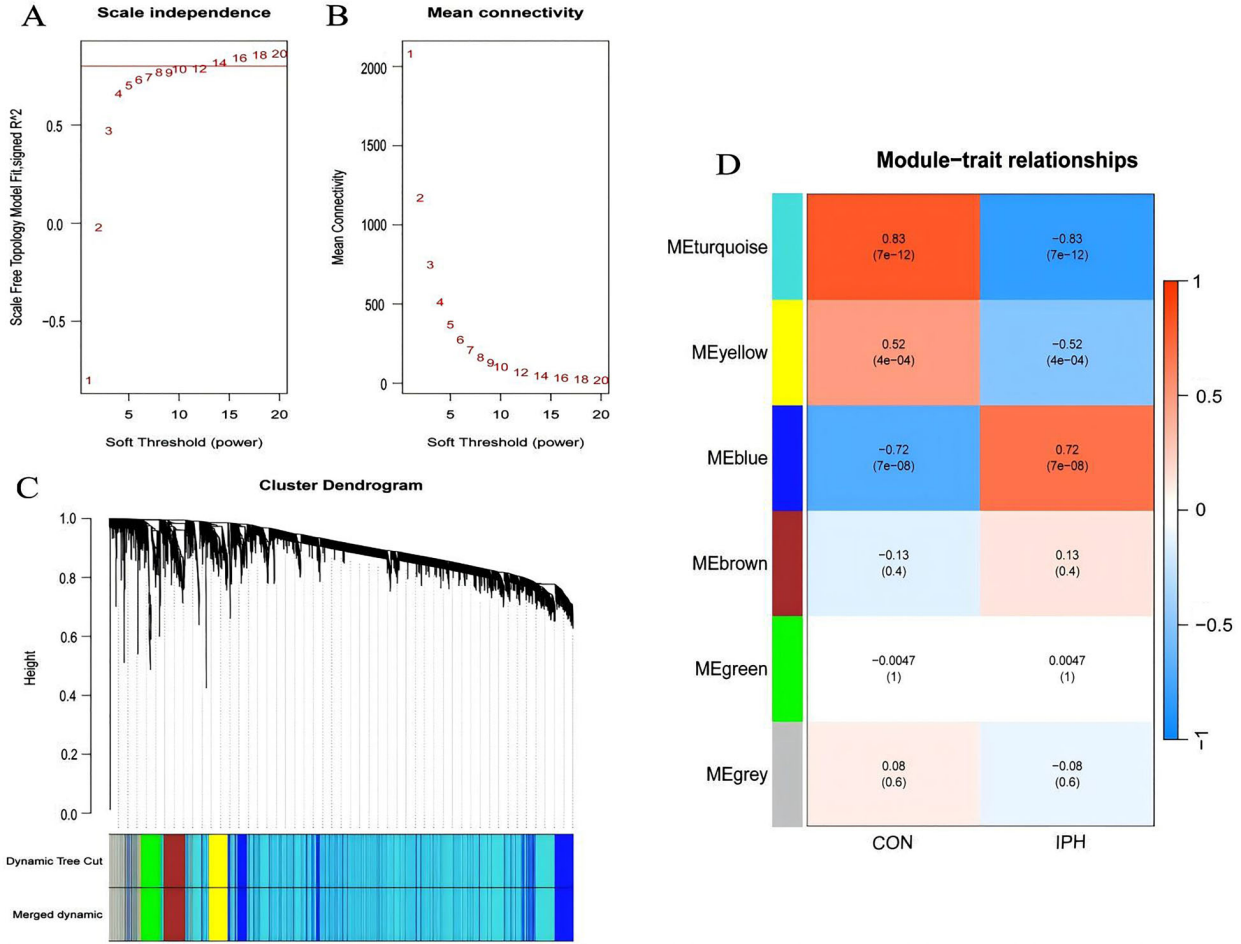
Weighted gene co-expression network analysis (WGCNA). **A** and **B**, b=10 was chosen as the soft threshold based on scale independence and average connectivity. **C**, Gene co-expression modules with different colors under the gene tree. **D**, Module-trait relationships in intraplaque hemorrhage (IPH). Each cell contains the corresponding correlation and P-value.

Table 1 provides a summary of the four datasets, presenting the source of the dataset, platform, histopathological category, sample size, and category of this study. Figure 1 depicts a comprehensive flow chart illustrating the study process. A scale-free co-expression network was constructed using WGCNA to identify the modules that exhibited the strongest association with IPH in GSE163154. A “soft” threshold (b=10) was determined by considering scale independence and average connectivity (Figure 2A and B). This threshold was used to construct a dendrogram of IPH and control samples, resulting in the identification of 6 gene co-expression modules represented by different colors (Figure 2C). The module merging threshold was set at 0.25, with a minimum module size of 100. The results of the clinical correlation analysis revealed that the turquoise, yellow, and blue modules exhibited the strongest correlation with IPH (turquoise module: r =-0.83, P=7e-12; yellow module: r=-0.52, P=4e-04; blue module: r=0.72, P=7e-08). The three modules displayed significant correlations with IPH and consisted of 4309 genes (Figure 2D). Consequently, we chose to further analyze the three modules.

**Table 1:** Details of the four datasets analyzed in the study.

ID	Data set source	GSE number	Platform	Histopathological category and sample size	Category of this study
1	GEO- human	GSE163154	GPL6104	27 IPH and 16 non-IPH	training set
2	GEO- human	GSE28829	GPL570	16 advanced AS plaques and 13 early AS plaques	validation set
3	GEO- human	GSE41571	GPL570	5 ruptured plaques and 6 stable plaques	validation set
4	GEO- human	GSE120521	GPL16791	4 unstable plaques and 4 stable plaques	validation set
5	GEO- human	Merged set of the four datasets		52 disease and 39 control	validation set

GEO: Gene Expression Omnibus.

### Functional annotation and pathway enrichment of NRGs

**Figure 3:**
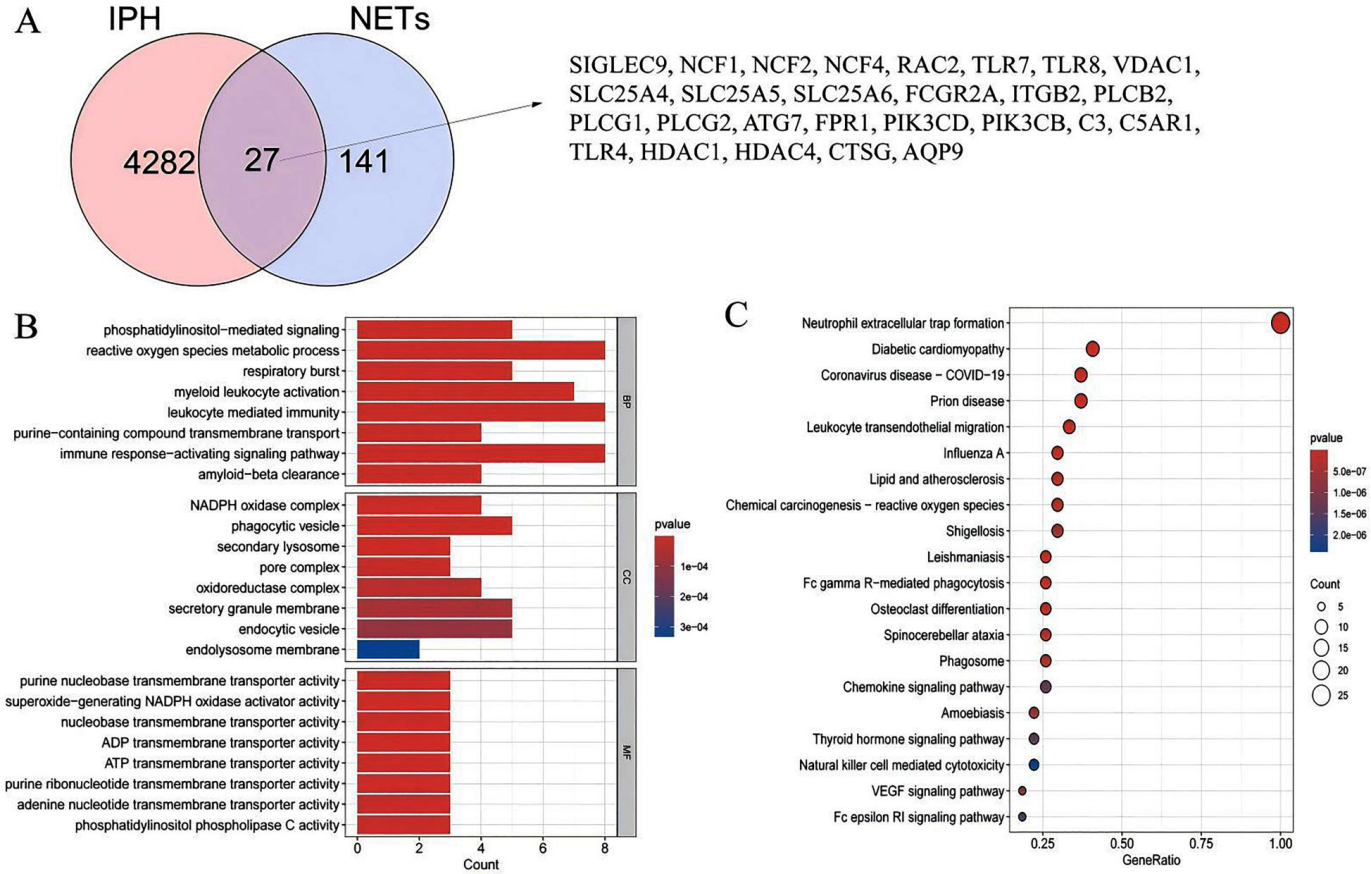
Acquisition and enrichment analysis of communal genes. **A**, Overlap of communal genes between the NETs-related genes (NRGs) and three modules of IPH. **B**, Gene Ontology (GO enrichment analyses of communal genes. **C**, Kyoto Encyclopedia of Genes and Genomes (KEGG) pathway enrichment analysis of communal genes. IPH: intraplaque hemorrhage; NETs: neutrophil extracellular traps.

We crossed the three significant modules obtained from WGCNA analysis with NRGs and identified a total of 27 communal genes. Their overlap was visually represented using Venn diagrams (Figure 3A). Subsequently, we performed GO and KEGG enrichment analyses on the set of 27 communal genes using the “clusterProfiler” R package (Figure 3B and C). The enrichment results of the communal genes were primarily ranked based on the P-values of the three functional components (Table 2). The GO-BP analysis revealed significant involvement of the communal genes in the phosphatidylinositol-mediated signaling, reactive oxygen species metabolic process, respiratory burst, myeloid leukocyte activation, leukocyte mediated immunity, purine-containing compound transmembrane transport, immune response-activating signaling pathway, and amyloid-beta clearance. The GO-CC analysis primarily highlighted the involvement of the communal genes in NADPH oxidase complex, phagocytic vesicle, secondary lysosome, pore complex, oxidoreductase complex, secretory granule membrane, endocytic vesicle, and endolysosome membrane. The key findings from the GO-MF enrichment analysis revealed the involvement of the communal genes in various molecular functions, including purine nucleobase transmembrane transporter activity, superoxide-generating NADPH oxidase activator activity, nucleobase transmembrane transporter activity, ADP transmembrane transporter activity, ATP transmembrane transporter activity, purine ribonucleotide transmembrane transporter activity, adenine nucleotide transmembrane transporter activity, and phosphatidylinositol phospholipase C activity. Furthermore, KEGG pathway analysis revealed significant enrichment of communal genes in pathways such as neutrophil extracellular trap formation, leukocyte transendothelial migration, Fc gamma R-mediated phagocytosis, chemokine signaling pathway, thyroid hormone signaling pathway, VEGF signaling pathway, and Fc epsilon RI signaling pathway.

**Table 2:** Enrichment analysis of communal genes.

Category	ID	Description	Gene ID
GO -BP	GO: 0048015	phosphatidylinositol-mediated signaling	PLCB2/PLCG1/PLCG2/PIK3CD/PIK3CB
GO -BP	GO: 0072593	reactive oxygen species metabolic process	NCF1/NCF2/NCF4/RAC2/VDAC1/ITGB2/PLCG2/TLR4
GO -BP	GO: 0045730	respiratory burst	NCF1/NCF2/NCF4/RAC2/PIK3CD
GO -BP	GO: 0002274	myeloid leukocyte activation	RAC2/ITGB2/PLCG2/PIK3CD/C5AR1/TLR4/CTSG
GO -BP	GO: 0002443	leukocyte mediated immunity	RAC2/TLR8/ITGB2/PLCG2/PIK3CD/C3/TLR4/CTSG
GO -BP	GO: 0072530	purine-containing compound transmembrane transport	SLC25A4/SLC25A5/SLC25A6/AQP9
GO -BP	GO: 0002757	immune response-activating signaling pathway	TLR7/TLR8/PLCG1/PLCG2/FPR1/PIK3CD/C5AR1/TLR4
GO -BP	GO: 0097242	amyloid-beta clearance	ITGB2/C3/C5AR1/HDAC1
GO -CC	GO: 0043020	NADPH oxidase complex	NCF1/NCF2/NCF4/RAC2
GO -CC	GO: 0045335	phagocytic vesicle	NCF1/NCF2/NCF4/RAC2/TLR7
GO -CC	GO: 0005767	secondary lysosome	NCF1/NCF2/NCF4
GO -CC	GO: 0046930	pore complex	VDAC1/SLC25A4/SLC25A5
GO -CC	GO: 1990204	oxidoreductase complex	NCF1/NCF2/NCF4/RAC2
GO -CC	GO: 0030667	secretory granule membrane	SIGLEC9/FCGR2A/ITGB2/FPR1/C5AR1
GO -CC	GO: 0030139	endocytic vesicle	NCF1/NCF2/NCF4/RAC2/TLR7
GO -CC	GO: 0036020	endolysosome membrane	TLR7/TLR8
GO -MF	GO: 0005345	purine nucleobase transmembrane transporter activity	SLC25A4/SLC25A5/AQP9
GO -MF	GO: 0016176	superoxide-generating NADPH oxidase activator activity	NCF1/NCF2/NCF4
GO -MF	GO: 0015205	nucleobase transmembrane transporter activity	SLC25A4/SLC25A5/AQP9
GO -MF	GO: 0015217	ADP transmembrane transporter activity	SLC25A4/SLC25A5/SLC25A6
GO -MF	GO: 0005347	ATP transmembrane transporter activity	SLC25A4/SLC25A5/SLC25A6
GO -MF	GO: 0005346	purine ribonucleotide transmembrane transporter activity	SLC25A4/SLC25A5/SLC25A6
GO -MF	GO: 0000295	adenine nucleotide transmembrane transporter activity	SLC25A4/SLC25A5/SLC25A6
GO -MF	GO: 0004435	phosphatidylinositol phospholipase C activity	PLCB2/PLCG1/PLCG2
KEGG	hsa04613	Neutrophil extracellular trap formation	SIGLEC9/NCF1/NCF2/NCF4/RAC2/TLR7/TLR8/VDAC1/SLC25A4/SLC25A5/SLC25A6/FCGR2A/ITGB2/PLCB2/PLCG1/PLCG2/ATG7/FPR1/PIK3CD/PIK3CB/C3/C5AR1/TLR4/HDAC1/HDAC4/CTSG/AQP9
KEGG	hsa04666	Fc gamma R-mediated phagocytosis	NCF1/RAC2/FCGR2A/PLCG1/PLCG2/PIK3CD/PIK3CB
KEGG	hsa04145	Phagosome	NCF1/NCF2/NCF4/FCGR2A/ITGB2/C3/TLR4
KEGG	hsa04370	VEGF signaling pathway	RAC2/PLCG1/PLCG2/PIK3CD/PIK3CB
KEGG	hsa04062	Chemokine signaling pathway	NCF1/RAC2/PLCB2/PLCG1/PLCG2/PIK3CD/PIK3CB
KEGG	hsa04919	Thyroid hormone signaling pathway	PLCB2/PLCG1/PLCG2/PIK3CD/PIK3CB/HDAC1
KEGG	hsa04664	Fc epsilon RI signaling pathway	RAC2/PLCG1/PLCG2/PIK3CD/PIK3CB

GO: Gene Ontology; CC: Cellular component; MF: molecular function; BP: biological process; KEGG: Kyoto Encyclopedia of Genes and Genomes.

### Identification of NETs-related hub genes

**Figure 4:**
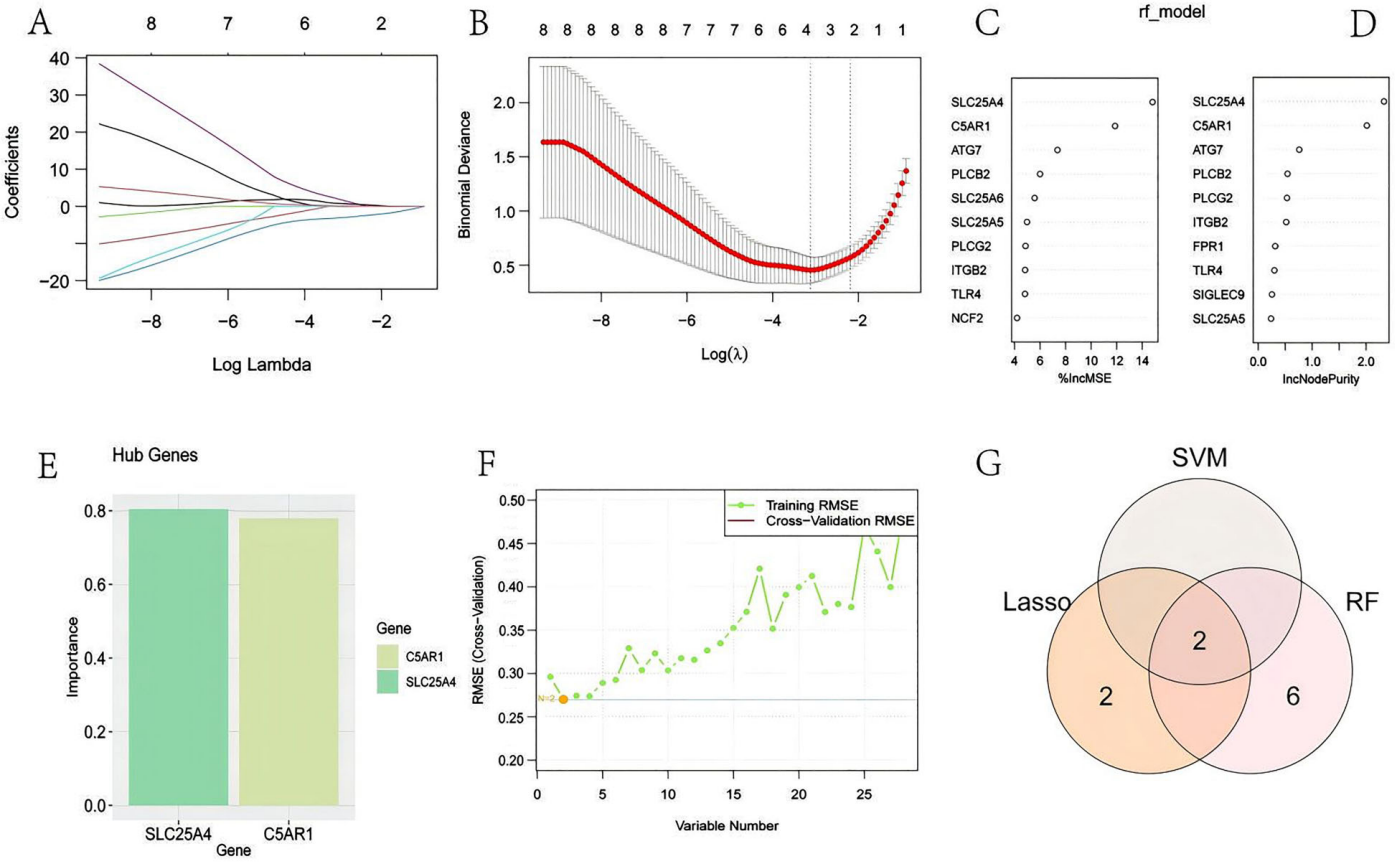
Machine learning for the identification of hub genes. **A** and **B**, Candidate hub genes identified in the least absolute shrinkage and selection operator (LASSO) model. **C** and **D**, Rank of the top 10 most important genes by the random forest (rf) algorithm based on %lncMSE and IncNodePurity. **E** and **F**, The root mean squared error (RMSE) of candidate hub genes combination of the support vector machine (SVM) algorithm. **G**, Venn diagram showing the overlap of candidate genes among the three algorithms.

LASSO regression, SVM, and RF machine learning algorithms were utilized to identify potential candidate genes associated with the progression of AS among communal genes. In the LASSO logistic regression genes, namely SLC25A4, SLC25A6, C5AR1, and CTSG, were identified as candidate hub genes based on the optimal lambda min value of 0.04427903 (Figure 4A and B). Lambda min is the lambda value corresponding to the smallest cross-validation error. Based on the % significance of communal genes in lncMSE and IncNodePurity, the RF classifier identified the top 10 overlapping genes as candidate hub genes, including SLC25A4, C5AR1, ATG7, PLCB2, SLC25A5, among others (Figure 4C and D). Additionally, two candidate genes (C5AR1 and SLC25A4) were identified when the classification error of the SVM model was lowest (Figure 4E and F). Table 3 provides a summary of the candidate genes identified by the three algorithms, while Figure 4G presents the visual representation of the algorithm results using Venn diagrams. Ultimately, SLC25A4 and C5AR1, which were identified as overlapping genes across all three algorithms, were selected as the hub genes.

**Table 3:** Candidate genes screened by the three algorithms.

Method	Gene
LASSO	SLC25A4 / SLC25A6 / C5AR1 / CTSG
SVM	C5AR1 / SLC25A4
RF	SLC25A4 / C5AR1 / ATG7 / PLCB2 / SLC25A5 / PLCG2 / ITGB2 / TLR4

LASSO: least absolute shrinkage and selection operator; SVM: support vector machine; RF: random forest.

### PPI and co-expression network construction

**Figure 5:**
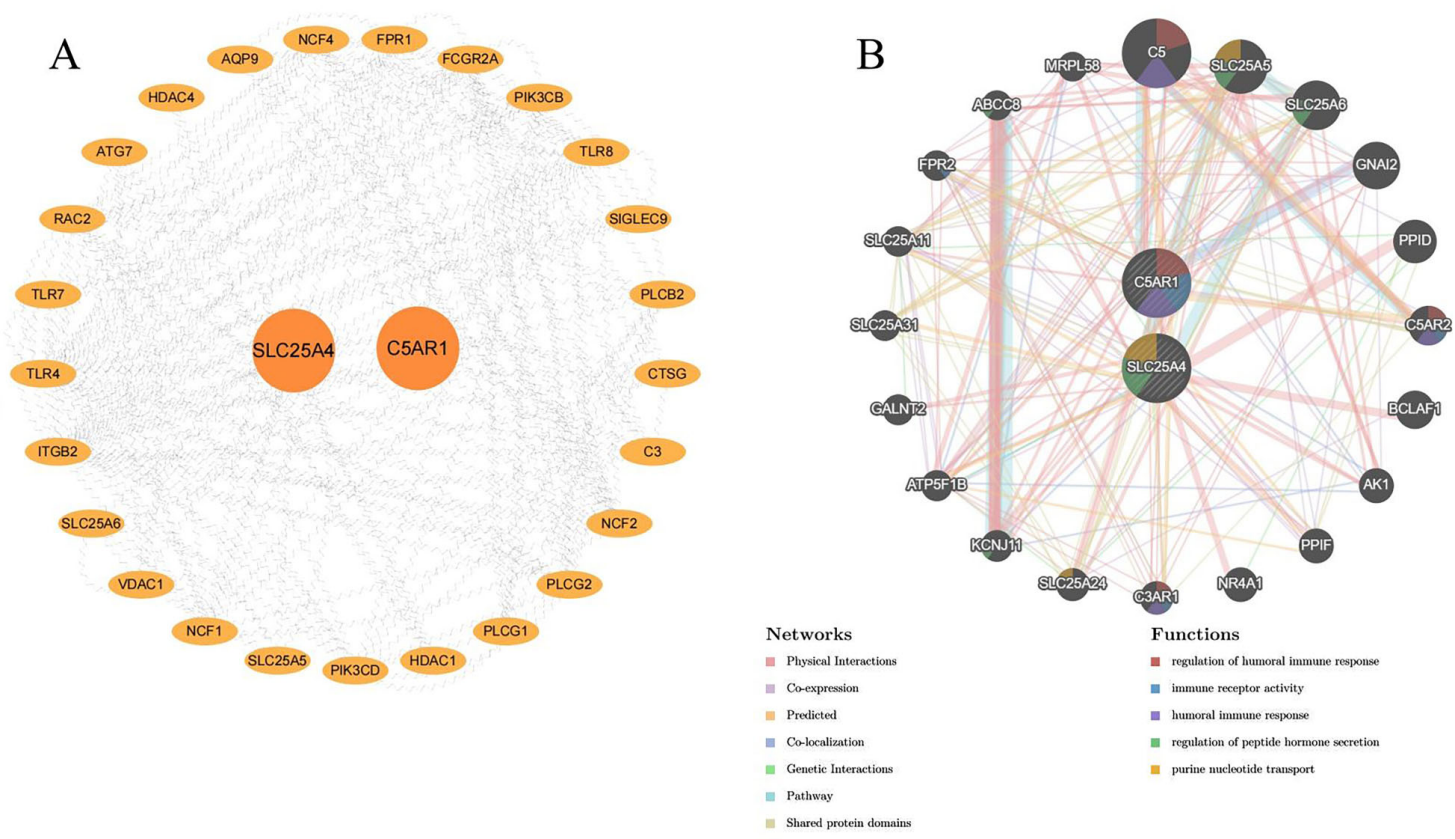
**A**, Protein-protein interaction (PPI) network analysis of communal genes and two hub genes. **B**, Co-expression network analysis of two hub genes by Genemania.

Following the hub genes screening described above, we conducted PPI network analysis for the communal genes and the two hub genes (Figure 5A). Furthermore, we utilized the GeneMANIA platform to construct a co-expression network for SLC25A4 and C5AR1 (Figure 5B). The PPI network analysis revealed that the two hub genes exhibited a complex network, comprising 77.64% physical interactions, 8.01% co-expression, 5.37% predicted interactions, 3.63% co-localization, 2.87% genetic interactions, 1.88% pathway associations, and 0.60% shared protein domains. The biological function and significance of the hub genes reinforce the importance of immunoregulation in IPH.

### Construction of a prediction model

**Figure 6:**
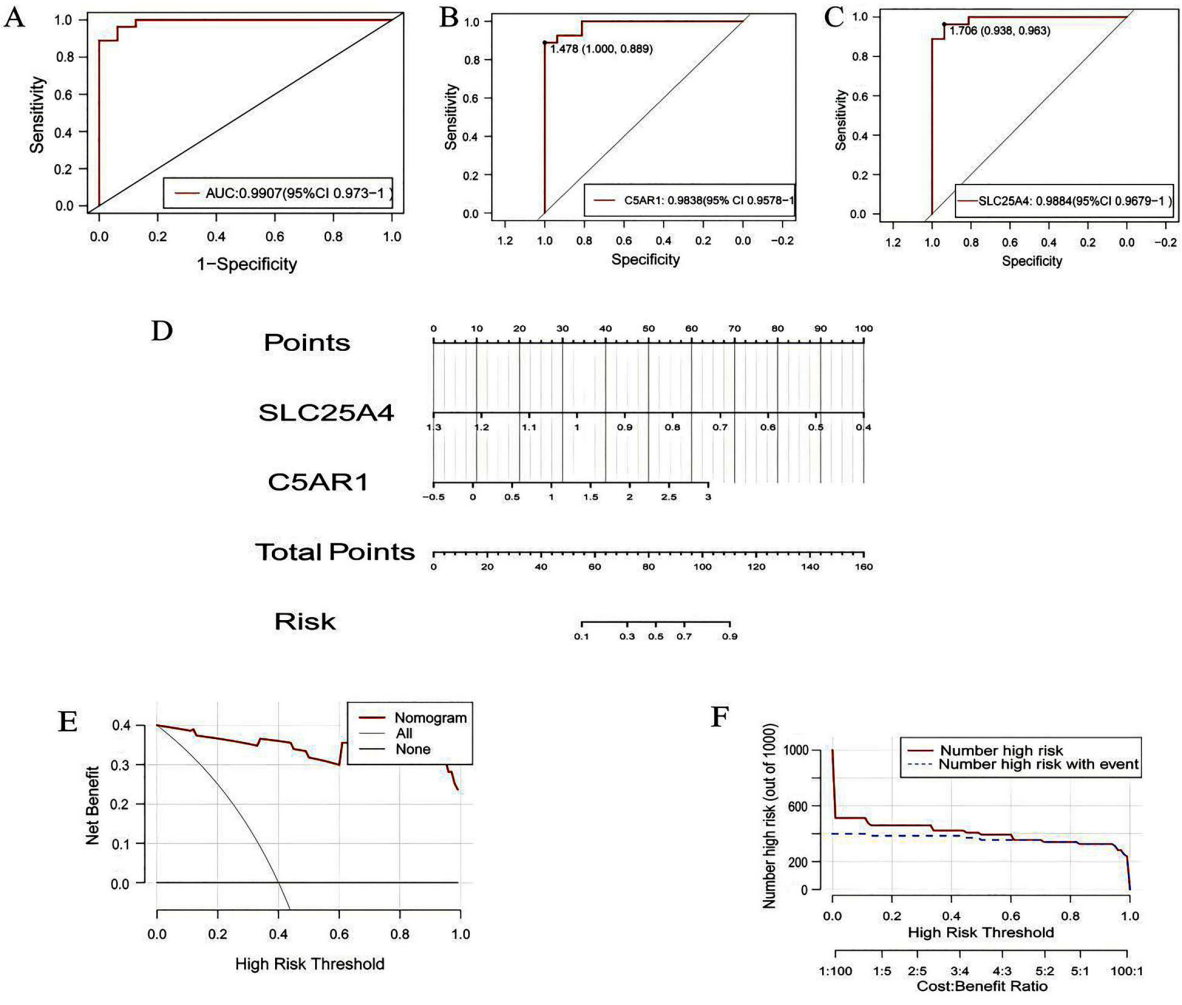
Performance of hub genes in the training set (GSE163154). **A**, ROC curve of the two hub genes combined diagnosis of intraplaque hemorrhage (IPH). **B** and **C**, ROC curve of SLC25A4 and C5AR1, respectively. **D**, Nomogram of the two hub genes. **E**, Decision curve analysis (DCA) of the prediction model. **F**, Clinical impact curve (CIC) of the prediction model.

Utilizing the two NETs-related hub genes, we developed a nomogram model to assess the probability of IPH and evaluated its predictive performance. The combined ROC analysis of the two hub genes achieved an area under the AUC of 0.9907 (Figure 6A), indicating excellent prediction performance. Furthermore, the individual AUCs for SLC25A4 and C5AR1 were 0.9884 and 0.9838, respectively, highlighting their potential as valuable predictive biomarkers (Figure 6B and C). Figure 6D illustrates the “total number of points”, and subsequently, DCA and CIC were used to evaluate the accuracy and utility of the diagnostic model, the results of which showed good prediction (Figure 6E and F).

### Validation of prediction models

**Figure 7:**
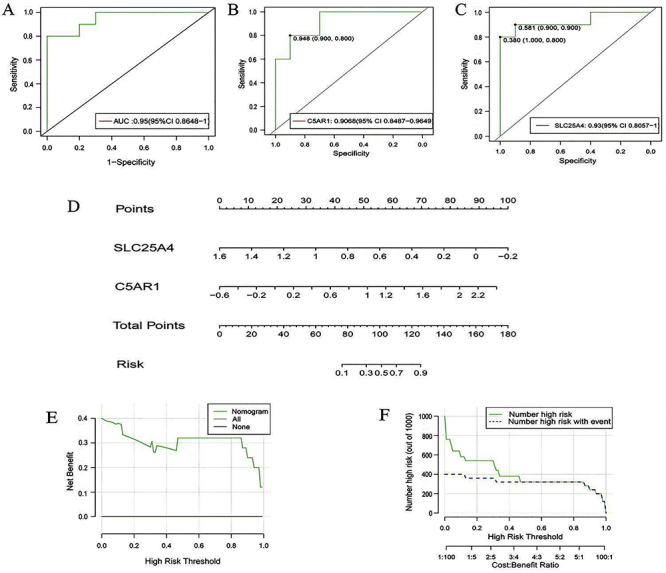
Validation of random representative samples of four data sets (GSE163154, GSE28829, GSE41571, and GSE120521). **A**-**C**, ROC curve of the two hub genes. **D**, Nomogram of the two hub genes. **E**, Decision Curve Analysis (DCA) of the validation model. **F**, Clinical impact curve (CIC) of the validation model.

Additionally, we combined four data sets (GSE163154, GSE28829, GSE41571, GSE120521) and conducted random sampling validation. The combined ROC analysis of the two hub genes achieved an AUC of 0.95 (Figure 7A), also indicating excellent prediction performance. Furthermore, the individual AUCs for C5AR1 and SLC25A4 were 0.9068 and 0.93 (Figure 7B and C). Figure 7D illustrates the “total number of points”, and we used DCA and CIC to evaluate the accuracy and utility of the diagnostic model again, which showed good prediction results (Figure 7E and F). These three datasets (GSE28829, GSE41571, GSE120521) independently validated the results.

### Immune infiltration analysis

**Figure 8:**
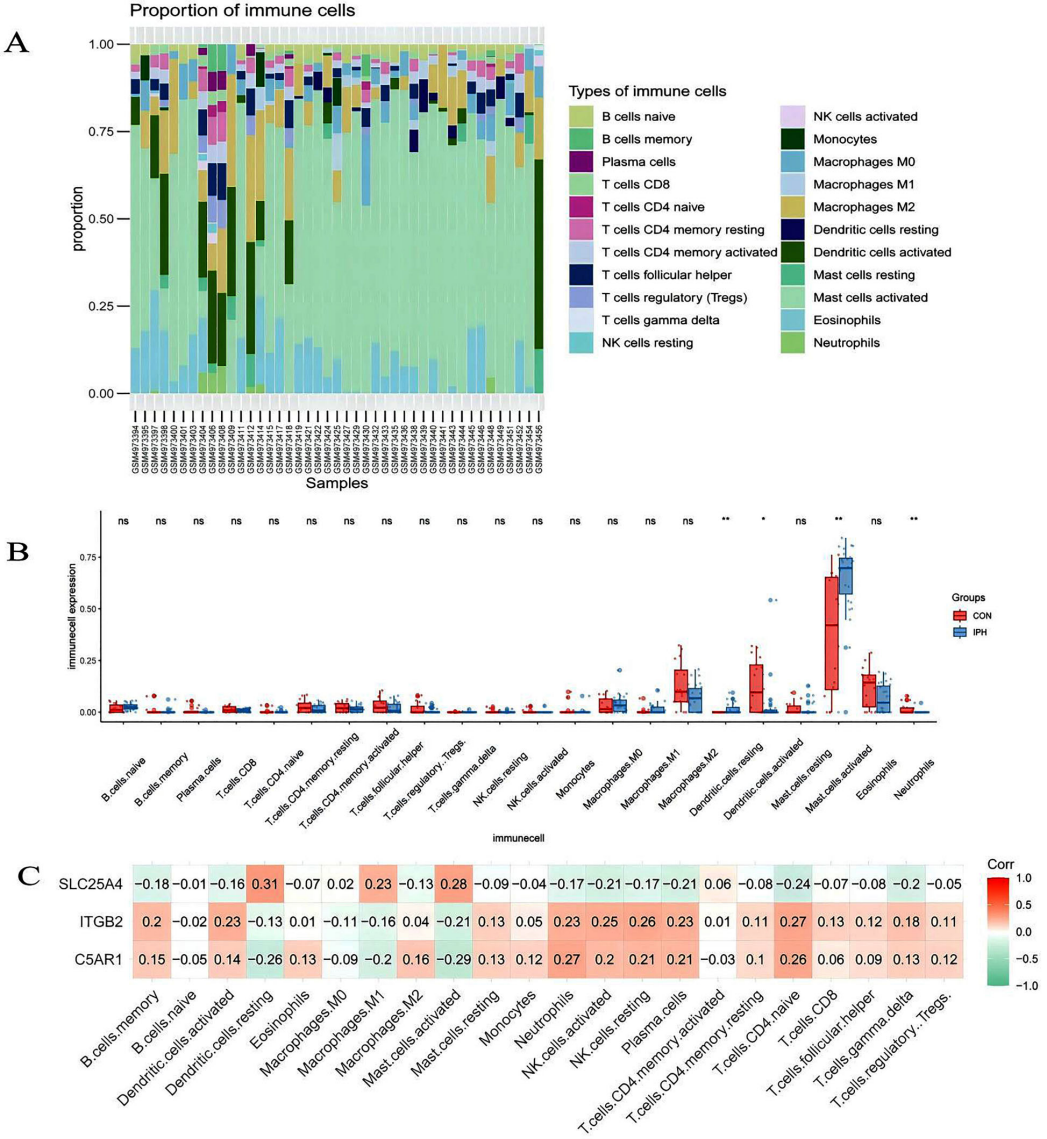
Immune cell infiltration comparison between intraplaque hemorrhage (IPH) and control. **A**, Proportion of 22 immunocytes in all samples. **B**, Comparison of the proportion of 22 types of immunocytes between IPH and control groups. **C**, Correlation heat map between hub genes and immune cells. *0.01<P<0.05, **0.001<P<0.01, ns: 0.05<P<1 (chi-squared test).

The enrichment of immune-related functions in the communal genes and hub genes were identified in our study. The proportion of 22 immunocytes in all samples are shown in the bar plot (Figure 8A). Figure 8B displays the relative proportions of 22 immune cell types in the IPH and control groups. The boxplot analysis revealed elevated levels of activated mast cells and resting dendritic cells in the IPH group compared to the control group. Neutrophils and activated dendritic cells were observed at lower levels in the IPH group.

### Correlation analysis of hub genes and immune cells

Correlation analysis between the two hub genes and infiltrating immune cells (Figure 8C) revealed that SLC25A4 exhibited positive correlations with resting dendritic cells. In contrast, C5AR1 did not show a significant association.

Comprehensive analysis of immune infiltration results indicated that resting dendritic cells, activated mast cells, and activated dendritic cells may contribute to the immune processes and mechanisms involved in IPH.

### Real-time quantitative polymerase chain reaction

**Figure 9:**
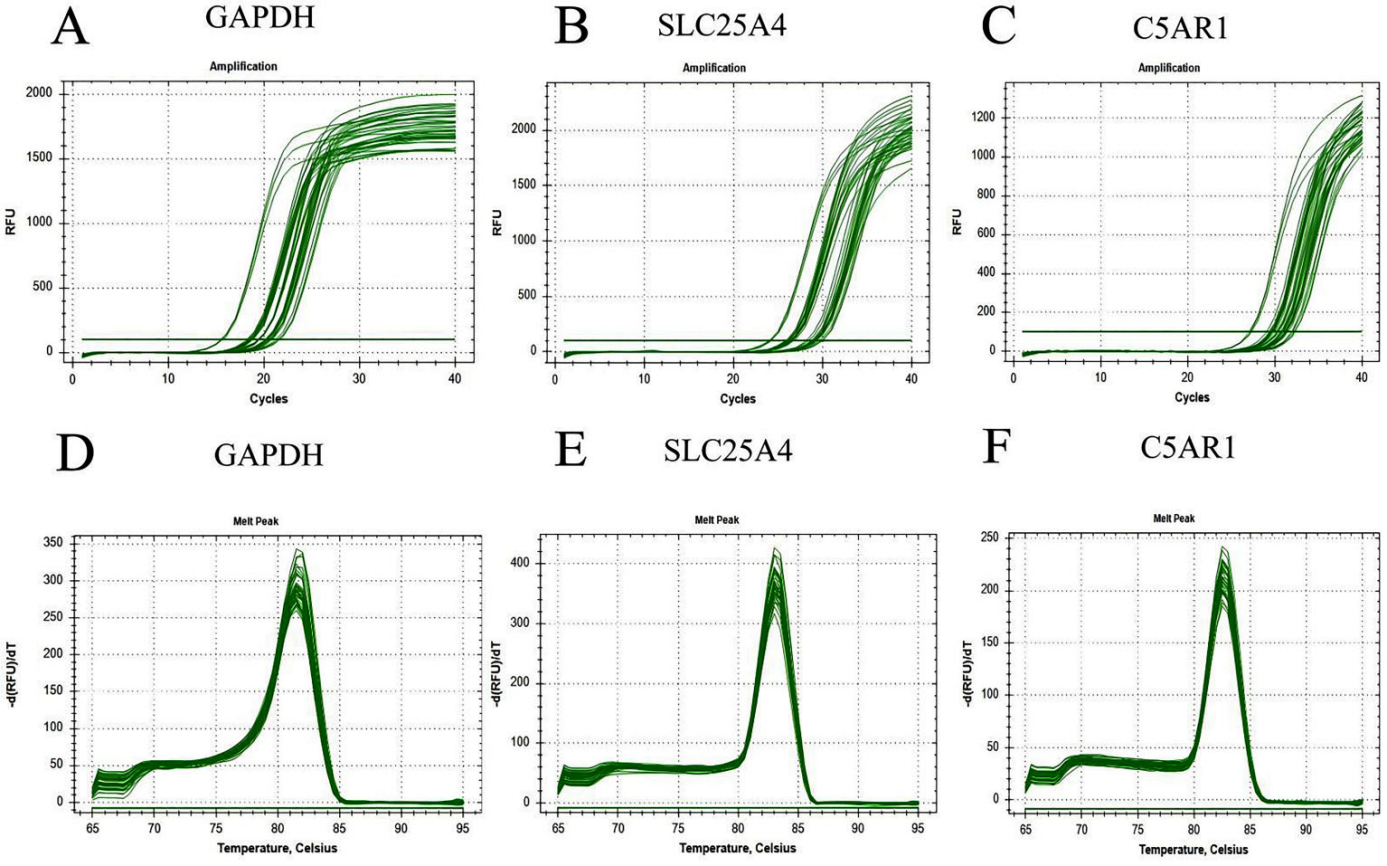
**A**-**C**, Amplification curves of the reference gene, SLC25A4, and C5AR1. **D**-**F**, Dissolution curve of the reference gene, SLC25A4, and C5AR1.

**Figure 10:**
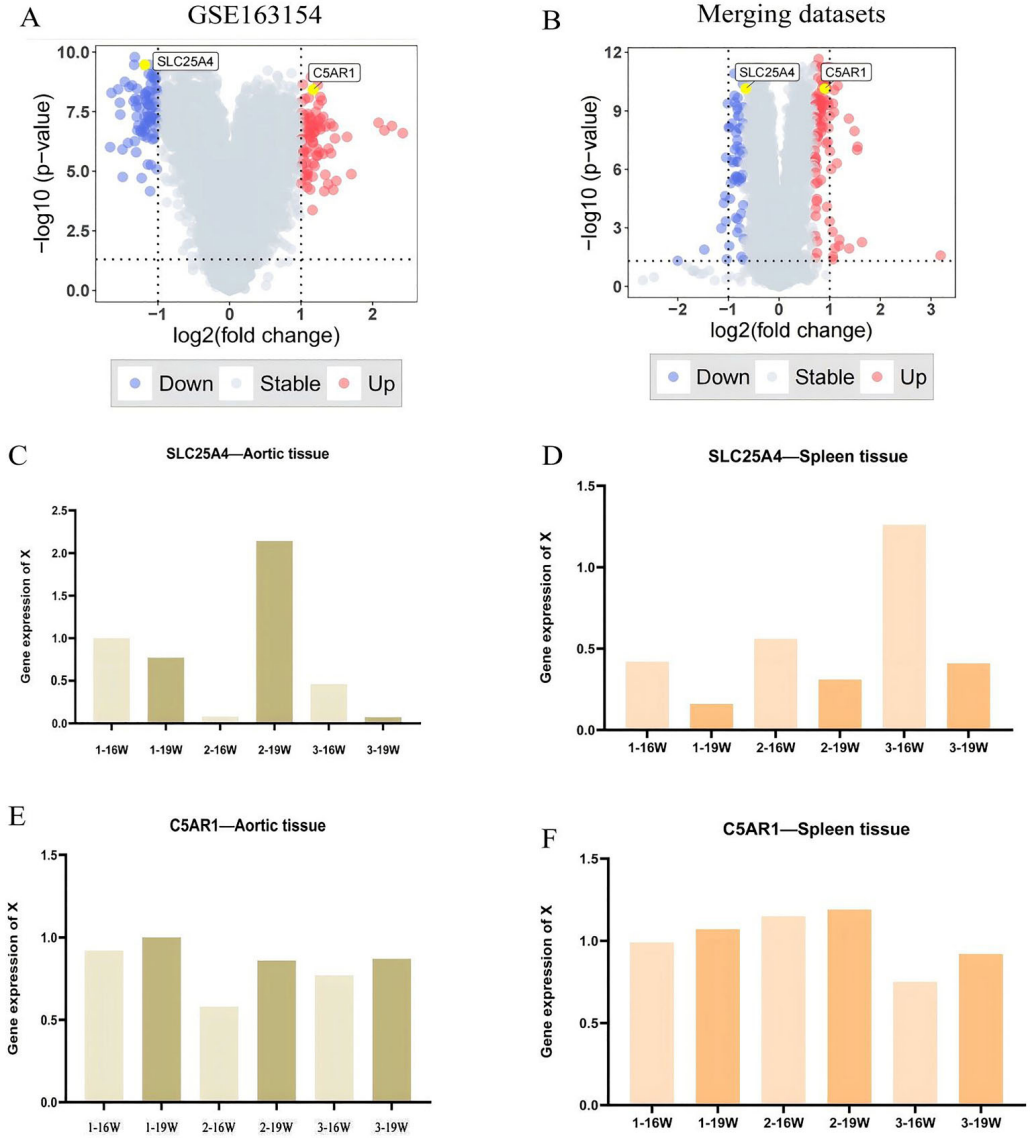
Expression trends of the two hub genes. **A** and **B**, Volcano plot of gene expression in GSE163154 and merging datasets. **C** and **D**, SLC25A4 expression in the aorta and spleen of three mice. **E** and **F**, C5AR1 expression in the aorta and spleen of three mice.

To validate the reliability of the above datasets and the significance of hub genes, we further assessed the expression levels of two hub genes in atherosclerotic mice samples using qRT-PCR. Figure 9 shows the amplification and dissolution curves of the genes. The experimental results were generally similar to those obtained from mRNA microarrays in samples related to AS progression, exhibiting consistent expression trends (Figure 10A and B). Compared to early-stage (16-week) samples, the expression level of SLC25A4 in late-stage (19-week) atherosclerotic mice exhibited a decreasing trend (Figure 10C and D). Conversely, the expression level of C5AR1 in late-stage (19-week) atherosclerotic mice demonstrated an increasing trend compared to early-stage (16-week) samples (Figure 10E and F).

## Discussion

Due to limitations in time, resources, or specific conditions, the sample size included in this study was relatively small. However, the experimental design and methods followed the best scientific principles and practices to ensure the accuracy and reliability of the results. The sources, processing, and storage conditions of the six samples were consistent. Regarding the abnormally high expression level of SLC25A4 in the aortic arch tissue of the second group of mouse samples, we speculated that this may be related to heterogeneous genetics. Genetic heterogeneity refers to the phenomenon where a genetic trait can be caused by multiple different genetic alterations. Different cells or tissues may exhibit different gene expression patterns, which may lead to inconsistent PCR results.

The primary function of DCs is to initiate antigen-specific adaptive immune responses while maintaining self-antigen tolerance. In mice and healthy young individuals, DCs are located within the arterial intima, particularly in areas predisposed to atherosclerosis, and accumulate abundantly in atherosclerotic lesions (34). Although animal and human studies have demonstrated that DCs can regulate cholesterol metabolism and lipid uptake in addition to modulating adaptive immune responses, the exact molecular mechanisms underlying the involvement of DCs in the development and progression of atherosclerosis remain elusive (35).

SLC25A4 belongs to the solute carrier family of proteins and encodes the mitochondrial ADP/ATP carrier (AAC1). This carrier plays a crucial role in transporting ATP and ADP between the mitochondrial matrix and cytoplasmic matrix, thereby ensuring the proper functioning of the respiratory chain (36). However, the exact mechanism by which SLC25A4 contributes to the progression of AS remains unclear, and relevant research in this area is scarce. In contrast, our study identified SLC25A4 as a key gene involved in the progression of AS. Through enrichment analysis, we observed that SLC25A4 is predominantly involved in the transmembrane transport of purine-containing compounds and nucleotides, ADP transport, as well as processes related to mitophagy, negative regulation of mitochondrial membrane permeability, and the formation of neutrophil extracellular traps (NETs). Previous studies have demonstrated the essential role of adenine nucleotide translocase (ANT), an essential gene for mitophagy *in vivo*, in the process (36). Moreover, SLC25A4 (ANT1) is one of the subtypes of ANT. Additionally, autophagy has been implicated in neutrophil differentiation and its key functions, such as degranulation, production of reactive oxygen species, and release of NETs (37). Based on these findings, we hypothesized that SLC25A4 may play a role in inducing the formation of NETs and potentially contribute to the acceleration of atherosclerosis by regulating mitophagy. However, the precise mechanisms underlying this phenomenon require further investigation.

Recent studies have consistently demonstrated the elevated expression of C5AR1 in unstable plaques compared to stable plaques, which aligns with our findings. C5AR1, expressed by monocytes and macrophages, plays a crucial role in cell-autonomous intracellular production of C5a, contributing significantly to potentially sterile inflammation in HFD-driven atherosclerotic disease. The effects of C5AR1 on IL-1β can vary depending on the nature and location of the inflammation trigger (38). Activation of C5AR1 on endothelial cells during enhanced complement activation triggers an inflammatory state, while activation of C5AR1 on innate immune cells promotes their attachment to inflamed endothelium, facilitates antigen uptake and tissue infiltration, and induces a proinflammatory effector phenotype (39). Collectively, these events contribute to the maintenance of chronic pathological tissue inflammation. Previous studies have confirmed that treatment of plaques with the cell-permeable C5AR1 inhibitor JPE1375 leads to a significant reduction in IL-1β production (38).

## Conclusions

In this study, we successfully identified two hub genes (SLC25A4 and C5AR1) associated with NETs using bioinformatics analysis and machine learning algorithms. These genes may be potential candidates for AS progression prediction and treatment. Additionally, we conducted a correlation analysis of immune infiltration in a dataset related to AS progression (IPH-GSE163154) and developed a predictive nomogram for AS progression to provide reference for further research in prediction and treatment of AS progression. Our study is a pioneering investigation to delve into the molecular characteristics of NRGs in the progression of AS. Our findings offer novel insights into the underlying mechanisms and potential therapeutic directions for AS. Nevertheless, larger sample sizes are necessary for further validation in bioinformatics analyses and experimental studies in animals or humans. We will also work on this and conduct further in-depth research in order to substantiate the clinical relevance of these genes in the context of AS progression.

## Supplementary Material

Click to view [pdf].

## References

[1] Xu S, Pelisek J, Jin ZG (2018). Atherosclerosis is an epigenetic disease. Trends Endocrinol Metab.

[2] Kobiyama K, Ley K (2018). Atherosclerosis. Circ Res.

[3] Ahmadi A, Argulian E, Leipsic J, Newby DE, Narula J (2019). From subclinical atherosclerosis to plaque progression and acute coronary events: JACC State-Of-The-Art Review. J Am Coll Cardiol.

[4] van Dam-Nolen DHK, Truijman MTB, van der Kolk AG, Liem MI, Schreuder FHBM, Boersma E (2022). Carotid plaque characteristics predict recurrent ischemic stroke and TIA: The PARISK (Plaque At RISK) study. JACC Cardiovasc Imaging.

[5] Bos D, Arshi B, van den Bouwhuijsen QJA, Ikram MK, Selwaness M, Vernooij MW (2021). Atherosclerotic carotid plaque composition and incident stroke and coronary events. J Am Coll Cardiol.

[6] Brinkmann V, Zychlinsky A (2012). Neutrophil extracellular traps: is immunity the second function of chromatin?. J Cell Biol.

[7] Megens RTA, Vijayan S, Lievens D, Döring Y, van Zandvoort MAMJ, Grommes J (2012). Presence of luminal neutrophil extracellular traps in atherosclerosis. J Thromb Haemost.

[8] Döring Y, Manthey HD, Drechsler M, Lievens D, Megens RTA, Soehnlein O (2012). Auto-antigenic protein-DNA complexes stimulate plasmacytoid dendritic cells to promote atherosclerosis. Circulation.

[9] Silvestre-Roig C, Braster Q, Wichapong K, Lee EY, Teulon JM, Berrebeh N (2019). Externalized histone H4 orchestrates chronic inflammation by inducing lytic cell death. Nature.

[10] Franck G, Mawson TL, Folco EJ, Molinaro R, Ruvkun V, Engelbertsen D (2018). Roles of PAD4 and NETosis in experimental atherosclerosis and arterial injury: implications for superficial erosion. Circ Res.

[11] Yang X, Ma Y, Chen X, Zhu J, Xue W, Ning K (2023). Mechanisms of neutrophil extracellular trap in chronic inflammation of endothelium in atherosclerosis. Life Sci.

[12] Edgar R, Domrachev M, Lash AE (2002). Gene expression omnibus: NCBI gene expression and hybridization array data repository. Nucleic Acids Res.

[13] Barrett T, Wilhite SE, Ledoux P, Evangelista C, Kim IF, Tomashevsky M (2013). NCBI GEO: archive for functional genomics data sets - update. Nucleic Acids Res.

[14] KEGG: Kyoto Encyclopedia of Genes and Genomes https://www.kegg.jp/entry/hsa04613.

[15] Langfelder P, Horvath S (2008). WGCNA: an R package for weighted correlation network analysis. BMC Bioinfomatics.

[16] Yu G, Wang LG, Han Y, He QY (2012). clusterProfiler: an R package for comparing biological themes among gene clusters. OMICS.

[17] The Gene Ontology Consortium (2019). The Gene Ontology Resource: 20 years and still GOing strong. Nucleic Acids Res.

[18] Kanehisa M, Goto S (2000). KEGG: kyoto encyclopedia of genes and genomes. Nucleic Acids Res.

[19] Friedman J, Hastie T, Tibshirani R (2010). Regularization paths for generalized linear models via coordinate descent. J Stat Softw.

[20] Meyer D, Dimitriadou E, Hornik K, Weingessel A, Leisch F Misc Functions of the Department of Statistics (e1071), TU Wien2014.

[21] Kuhn M (2008). Building predictive models in R using the caret package. J Stat Software.

[22] Liaw A, Wiener MC (2007). Classification and regression by random forest.

[23] Tibshirani R (2018). Regression shrinkage and selection via the lasso. J R Stat Soc B.

[24] Gold C, Sollich P (2003). Model selection for support vector machine classification. Neuralcomputing.

[25] Wang H, Yang F, Luo Z (2016). An experimental study of the intrinsic stability of random forest variable importance measures. BMC Bioinformatics.

[26] Franceschini A, Szklarczyk D, Frankild S, Kuhn M, Simonovic M, Roth A (2013). STRING v9.1: protein-protein interaction networks, with increased coverage and integration. Nucleic Acids Res.

[27] Smoot ME, Ono K, Ruscheinski J, Wang PL, Ideker T (2011). Cytoscape 2.8: new features for data integration and network visualization. Bioinformatics.

[28] Warde-Farley D, Donaldson SL, Comes O, Zuberi K, Badrawi R, Chao P (2010). The GeneMANIA prediction server: biological network integration for gene prioritization and predicting gene function. Nucleic Acids Res.

[29] Harrell FE (2023). rms: Regression Modeling Strategies.

[30] Robin X, Turck N, Hainard A, Tiberti N, Lisacek F, Sanchez JC (2011). pROC: an open-source package for R and S+ to analyze and compare ROC curves. BMC Bioinformatics.

[31] Newman AM, Liu CL, Green MR, Gentles AJ, Feng W, Xu Y (2015). Robust enumeration of cell subsets from tissue expression profiles. Nat Methods.

[32] Patil I (2021). Visualizations with statistical details: the ‘ggstatsplot' approach. J Open Source Software.

[33] Wickham H (2016). ggplot2: Elegant graphics for data analysis.

[34] Zernecke A (2015). Dendritic cells in atherosclerosis: evidence in mice and humans. Arterioscler Thromb Vasc Biol.

[35] Kloc M, Kubiak JZ, Ghobrial RM (2022). Macrophage-, dendritic-, smooth muscle-, endothelium-, and stem cells-derived foam cells in atherosclerosis. Int J Mol Sci.

[36] Hoshino A, Wang WJ, Wada S, McDermott-Roe C, Evans CS, Gosis B (2019). The ADP/ATP translocase drives mitophagy independent of nucleotide exchange. Nature.

[37] Remijsen Q, Vanden Berghe T, Wirawan E, Asselbergh B, Parthoens E, De Rycke R (2011). Neutrophil extracellular trap cell death requires both autophagy and superoxide generation. Cell Res.

[38] Niyonzima N, Rahman J, Kunz N, West EE, Freiwald T, Desai JV (2021). Mitochondrial C5aR1 activity in macrophages controls IL-1β production underlying sterile inflammation. Sci Immunol.

[39] Reis ES, Mastellos DC, Hajishengallis G, Lambris JD (2019). New insights into the immune functions of complement. Nat Rev Immunol.

