# Supplementary material for: Establishment of a prediction model and immune infiltration characteristics of atherosclerosis progression based on neutrophil extracellular traps-related genes
Source: Braz J Med Biol Res. 2025 Mar 3;58:e13639. doi: 10.1590/1414-431X2024e13639 (PMC11884769; doi:10.1590/1414-431X2024e13639)
Supplement: Supplementary file 1 [file 1414-431X-bjmbr-58-e13639-suppl.pdf]

Table S1. List of the primers and primer sequences that were created by Sangon Biotech (China).

Primer information	Primer name	Primer sequence (5'-3')	Fragment length (bp)	Annealing temperature (℃)
NM_017008.4	GAPDH-S	CTGGAGAAACCTGCCAAGTATG	138	60
	GAPDH-A	GGTGGAAGAATGGGAGTTGCT		60
NM_053515.2	Slc25a4-S	GCTGCCTACTTCGGAGTCTATG	242	60
	Slc25a4-A	GCTTTGGCTCCTTCATCTTTT		60
NM_053619.2	C5AR1-S	TGATGGTGGGTTTCGTGTTG	107	60
	C5AR1-A	TTTGAGCGTCTTGGTGGAGC		60

Forward primer: -S (sense primer). Reverse primer: -A (antisense primer).
